# Investigating Primary School Children’s Creative Potential Through Dynamic Assessment

**DOI:** 10.3389/fpsyg.2019.00733

**Published:** 2019-04-05

**Authors:** Dimitrios Zbainos, Athanasia Tziona

**Affiliations:** Department of Home Economics and Ecology, Harokopio University, Athens, Greece

**Keywords:** creative potential, dynamic assessment, mediation, graduated prompting, primary education

## Abstract

This paper focused on examining primary school students’ creative potential (CP) through dynamic assessment (DA). The study was carried out through a quasi-experimental design. The sample consisted of 90 Greek primary school students between fourth and sixth grade who were randomly divided into a group that received DA (*N* = 37) and a control group (*N* = 53). Both groups were initially tested with the graphic-artistic form of the Evaluation of Potential Creativity (EPoC) test. The DA group received mediation with graduated prompting while no such treatment was applied to the control group, and both groups were post-tested. The results demonstrated that mediation significantly improved DA group’s CP. It appeared that DA contributes in demonstrating a clearer portrait of students’ CP which can be of valuable assistance for nurturing creativity.

## Introduction

Classroom assessment has been defined as a “process of collecting, synthesizing, and interpreting information to aid in decision making” (Russell and Airasian, 2011, p. 3). It has been characterized as a core element of teaching and learning (Newton, 2007; Russell and Airasian, 2011) while one of its core aims is to help students to reach their fullest potential (Jones, 2009).

Intellectual potential has traditionally been assessed by psychometric testing that claimed to measure the “g factor,” an unchangeable genetically predetermined ability (Jensen, 1998). Nevertheless, psychometric testing has not been proven to be very helpful in education as it does not provide adequate information about children’s learning processes and their levels of understanding (e.g., Haywood and Lidz, 2007; Grigorenko, 2009) on which differentiated instruction can be grounded (Fisher and Frey, 2015). It has also been claimed that psychometric testing even has a negative impact on some students’ learning (Stiggins, 2005; Black et al., 2010).

On the contrary, formative assessment or assessment for learning aims at unlocking and enhancing students’ potential by utilizing judgments about the quality of student performances and pieces, or works (Sadler, 1989; Black and Wiliam, 2009; Wiliam, 2011). However, formative assessment has received criticisms related to the absence of fundamental measurement principles and mostly to the lack of a solid psycho-educational theoretical background (Yorke, 2003; Bennett, 2011; Coffey et al., 2011).

### Dynamic Assessment

Dynamic assessment (DA) aims at revealing student potential systematically and based on a sound and valid theoretical background. It was defined as:

An umbrella term used to describe a heterogeneous range of approaches that are linked by a common element, that is, instruction and feedback are built into the testing process and are differentiated on the basis of an individual’s performance (Elliott, 2003, pp. 16–17).

Although the given definitions of DA may vary, their constant aspect is the active intervention by examiners and the assessment of examinees’ response to intervention (Haywood and Tzuriel, 2002). Theoretically DA is grounded in Vygotsky’s Sociocultural Theory of Mind (e.g., Vygotsky, 1978) and Feuerstein’s theory of structural cognitive modifiability (e.g., Feuerstein et al., 1979, 1981, 1991; Feuerstein and Jensen, 1980). The main focus of DA is to reveal the potential of the assessee. The term “potential” is endorsed by most DA theorists as described in the definition of zone of proximal development Vygotsky (1978) as:

The distance between the actual developmental level as determined by independent problem solving and the level of potential development as determined through problem-solving under adult guidance, or in collaboration with more capable peers (p. 86).

According to this definition learning potential is conceptualized by taking into account the expressed ability (level) of a student before and after guidance. Hence, the fundamental difference of DA compared to static normative assessment is its interest in the learning outcomes that occur through a process that involves guidance or mediation and not in a score provided by static measurement that might not be indicative of the assessees’ potential (Gustafson et al., 2014; Navarro and Lara, 2017). DA theorists do not completely deny that static normative tests provide evidence about students’ potential as they appear to have a predictive validity of school performance (Haywood and Lidz, 2007), but argue that such a manifestation may be fractional and biased especially against those from different cultural environments (Grigorenko, 2009) and with learning disabilities (Fletcher et al., 2018).

Since the early studies of DA and in line with the Vygotskian approach, the word mediation has been used to describe a form of intervention aiming at removing the barriers of learning and performance through the tailor made individual instructions and the interaction between the mediator and the child (Poehner, 2008). The word mediation is conceptually close to educational or training interventions for developing cognitive abilities, however, minor differences have been identified: Mediational is directed to the means of promoting logical thinking and systematic learning while intervention usually refers to academic learning and curriculum (Haywood and Lidz, 2007).

The various models of DA have employed approaches that differ in the sequence of testing and mediation takes as well as in the type of mediation. The most commonly used DA approach is the “sandwich procedure” with graduated prompting. According to “sandwich” procedure the examinees are assessed by a first test (pre-test), then they receive individual mediation adjusted to their personal needs, strengths and weaknesses, and afterwards they are assessed through a second test (post-test) (Sternberg and Grigorenko, 2002). The assessment’s result is estimated by taking into account both the pre-test and the post-test (Haywood and Lidz, 2007) or only the post-test (Sternberg and Grigorenko, 2002). The result therefore, is not a static estimation of the child’s cognitive abilities but a process that leads to the manifestation of the child’s cognitive potential through individually tailored mediation (Elliott, 2003). Graduated prompting, rooted in the pioneering work of Campione et al. (1985) is a widely applied mediational approach of DA (e.g., Poehner and Lantolf, 2013; Navarro and Lara, 2017; Resing et al., 2017a,b). In this approach the prompts are designed on the basis of each child’s learning needs and gradually reduced as the child’s ability to cope sufficiently and independently with the task demands raises (Resing, 2013).

The sandwich approach with graduated prompting has been primarily employed for the DA of inductive or analogical reasoning. Several studies have shown that in post-testing through this procedure children from different cultural environments expressed their potential in analogical thinking by employing more advanced strategies in problem solving (e.g., Resing et al., 2009, 2017a) as well as advanced verbal and behavioral inductive strategies in learning (e.g., Resing et al., 2017b). It has also been successfully applied for the DA of second language listening and reading comprehension (e.g., Poehner and Lantolf, 2013), for the DA of academically disadvantaged students’ reading difficulties (e.g., Navarro and Lara, 2017) and for the DA of mathematical problem solving (e.g., Wang, 2010).

To our knowledge DA has never been applied to the domain of creativity. Based on its effectiveness in revealing student’s potential in the domains mentioned above we presumed that the employment of DA in creativity would provide useful insights regarding creative potential (CP) and its development.

### Creative Potential and Its Measurement

Besides the lack of consensus among the researchers and theorists in defining creativity (Weisberg, 2015), it is commonly described as the ability to produce novel and context appropriate work (Kaufman and Sternberg, 2007; Beghetto and Kaufman, 2014). Although the development of creativity has been considered as one of the major goals of students’ learning (e.g., Rotherham and Willingham, 2010; Binkley et al., 2012; Wyse and Ferrari, 2015) still is generally accepted that there is a lot need to be done, since creativity expression at schools remains to a great extent an unachieved educational goal (Beghetto and Kaufman, 2014).

Creativity testing has followed the intelligence psychometric tradition; some divergent creative thinking batteries as Torrance (1972, 1988) test for creativity (TTCT) or Wallach and Kogan (1965) have been widely used for the assessment of creativity (Kaufman et al., 2008, 2012). Regardless of modern criticism of psychometric normative measurement in relation to their construct, content, predictive discriminant and ecological validity (for a review see Zeng et al., 2011), their creators claimed that they measured CP by assessing general abilities that under certain circumstances would be realized in later life, something which appears to be happening up to a degree (Kim, 2006; Runco et al., 2010).

Creative potential has been described as an existing, not yet expressed set of creative abilities that every student has, rather than outstanding creative performance (Runco, 2003). It has been defined as a latent ability to produce original, adaptive work, which is part of an individual’s “human capital” (Walberg, 1988). In recent years a systematic attempt to study and measure CP on a substantiated theoretical basis was attempted by Barbot et al. (2011, 2015, 2016) and Lubart et al. (2012, 2013). After an extensive review of the relevant literature and a significant number of studies they carried out, they proposed the confluence theory of CP according to which several distinct, but interrelated resources, such as biological and genetic factors, aspects of cognition like divergent thinking, aspects of conation (personality, motivational and emotional factors) and task-relevant knowledge are reflected in CP. They described that CP has been assessed either by resource-based (or analytic) approaches where the fit between an individual’s resources and creative task demands has been examined, or outcome-based (or “holistic”) approaches which include test tasks that resemble aspects of creative work.

On the basis of the confluence theory of CP, and having taken into account the existing approaches of CP measurement, they devised the “EPoC” test. EPoC explores the human resources described in the confluence theory through abstract and concrete divergent-explanatory and convergent-integrative creative thinking tasks. Divergent thinking tasks stimulate flexibility, divergent thinking, selective encoding (aspects of cognition), supported by openness to experiences and intrinsic task-oriented motivation (conative factors). Convergent thinking tasks stimulate associative thinking selective comparison and combination (aspects of cognition) supported by conative factors such as tolerance for ambiguity, perseverance, risk taking, and achievement motivation. Task relevant knowledge is also taken into account by including concrete tasks stimulated by known objects to the student as well by abstract thinking, where students are asked to apply existing knowledge to abstract stimulation and make it concrete (Lubart et al., 2012; Barbot et al., 2015, 2016). EPoC results can formulate multifaceted CP profiles which describe students’ strengths and weaknesses in divergent, convergent, abstract and concrete creative thinking processes of each domain (Lubart et al., 2012) which can be used as the basis for the design of teaching creativity programs (Barbot et al., 2015).

According to the confluence theory, CP is trainable as well as most of the individual resources involved in it. Although Lubart and colleagues have repeatedly demonstrated that education may affect the development of CP (e.g., Besançon and Lubart, 2008; Barbot et al., 2011; Besançon et al., 2013), they have stressed that more should be done in order to comprehend how it can be trained (Barbot et al., 2015). Thus it is implicitly accepted that a zone of proximal development of CP exists.

The main endeavor of the present study was to relate the confluence theory of CP to the Vygotskian perspective of cognitive development. It aimed to examine CP as described by the confluence theory in relation to the Vygotskian conceptualization of potential i.e., the development from unassisted to assisted expression of CP. It actually intended to study students’ “potential of CP” through DA; in other words to investigate the development of CP that mediation evokes. More specifically, it was hypothesized that both divergent-exploratory and convergent-integrative thinking as well as abstract and concrete thinking would develop significantly after mediation in accordance to the theory of DA and the relevant research findings.

## Materials and Methods

### Sample

The sample consisted of ninety students from the fourth fifth and sixth grades studying in two public neighboring elementary schools of a socioeconomically deprived inner city area of Athens in Greece (see Table 1). Students of the first school were used as the experimental (DA group, *N* = 37) group and students of the second school as the control group (*N* = 53). Their mean age was 11 years. Both participants and their parents consented to participate. The majority of participants (92%) were of Greek origin. According to their teachers’ reports participants of non-Greek descent had efficient language understanding and expression compared to the Greek students. In total, boys accounted for 49% and girls for 51% of the sample.

**Table 1:** Sample distribution by group, grade and gender.

	*N*	Percentage (%)		Frequency	Percentage (%)
DA group 4th grade	9	10%	Boys	6	67
			Girls	3	33
control group 4th grade	25	29%	Boys	11	44
			Girls	14	56
DA group 5th grade	11	12%	Boys	6	55
			Girls	5	45
control group 5th grade	18	20%	Boys	8	44
			Girls	10	56
DA group 6th grade	17	19%	Boys	10	58
			Girls	7	42
control group 6th grade	10	11%	Boys	3	30
			Girls	7	70

### Instrumentation

Creative thinking was assessed through the graphic – artistic version of EPoC (Lubart et al., 2012). EPoC provides two equivalent forms which can be used as pre- and post-tests for the measurement of interventions for creativity enhancement (Lubart et al., 2013). Both forms assess divergent explanatory thinking, through a graphic abstract and a concrete stimulus as well as convergent integrative thinking through a set of graphic abstract and concrete stimuli. The tasks are scored on a 7-point scale. EPoC is not standardized in Greece and therefore the French standardized scores were used as appear in the manual.

### Design and Procedure

In accordance to the Greek regulations, approval of the study was obtained by the Greek Ministry of Education acting on a proposal by the Institute of Educational Policy which examined the ethics and the methodology of the study (approval number 62671/Δ1/16/06-04-2017). The study was not approved by an Ethics Committee as this was not required as per applicable institutional and national guidelines. In accordance to the approval terms, first, the principals and teachers of the participating schools consented after they were informed about the purpose, the duration, the anonymity and the confidentiality of the study. Afterwards, a letter including a form of consent was send to the parents of the students including detailed information about the purpose of data collection, the duration of the study, as well as assurance for anonymity and confidentiality. In the letter it was also stressed that children would not run physical or psychological risks during the study and that they could withdraw at any point during the process. In this research only students whose parents signed the consent form participated.

A quasi-experimental design was implemented as more suitable to examine the effect of manipulative conditions to a specific group compared to the effect of its absence to another group (e.g., Cook et al., 2002; Seel, 2012) and hence it has been used in most DA studies as mentioned above. The study included three stages: (a) pre-testing of both DA and control group on a group – setting (b) individual mediation to the DA group students (c) post-testing of both groups on a group setting. Both pre- and post-testing were administered under the supervision of the researchers. Each test completion required approximately 40 min and students were allowed to ask questions in an unstressed and uncompetitive atmosphere.

The mediation was designed in accordance with the general procedural guidelines for conducting DA assessment as appear in Haywood and Lidz (2007) and executed in three steps for each task. Each step included graduated prompting adjusted and modified according to the individual responses of each child in pre-test tasks. It took place individually to every participant of the DA group in two phases, one for the abstract stimulation, and another for the concrete. Each phase required approximately 20 min.

The first step concerning the linguistic understanding of the requirements of each task consisted of three prompts used for all four tasks. It included the following prompts:

Prompt 1.1: Are there any words or phrases that you do not understand in the task instruction?
Prompt 1.2: Which words do you believe that will help you to think what to do?
Prompt 1.3: Can you repeat in your own words the task instruction?

The second step in the mediation aims at the acknowledgment of familiar concepts and ways of coping with the task demands. In divergent thinking it aimed at acknowledging students’ responses to the stimuli, and ways of producing new multiple ones. The prompts for creative divergent thinking tasks of EPoC were:

Prompt 2.1: Give a name for each one of your drawings.
Prompt 2.2: Can you put your drawings into groups? For example toys, fruit, etc.
Prompt 2.3: What other drawings can you make? Can you think of other groups for your drawings? Try to think of as many as you can

In convergent thinking the second step aimed at acknowledging the stimuli they chose to use, as well as to use more in original and integrative ways. The prompts for the convergent thinking tasks (second and forth) of EPoC were:

Prompt 2.1: Name the pictures of the card that you chose and sketch on your drawing. What did you draw?
Prompt 2.2: Do you think that you could use more pictures of the card? How could you use more pictures in your drawing?
Prompt 2.3: Can you think any other ideas that those pictures could be used in your drawing? Do you think that your drawing is different from those of your classmates?

The third step, aimed at the organization of thinking processes, techniques and methods. It included two prompts that were identical for all tasks while the third prompt was different for divergent and convergent tasks.

Prompt 3.1: Describe your thoughts for making these drawings before our discussion.
Prompt 3.2: After our discussion, do you think that there is another way to make your drawings? Describe how.

The last prompt for the divergent thinking tasks was:

Prompt 3.3: How do you think that you could draw as many as possible different drawings?

And the final prompt for convergent thinking tasks:

Prompt 3.4: How do you think that you could use and combine as many pictures as you can to your drawing and make it different from your classmates’?

During the mediation process students cooperated and actively interacted with the assessor.

## Results

### Initial Statistics

Table 2 shows the descriptive statistics of DA and control groups’ participants’ scores in the total pre-test and post-test of EPoC (Pre_Total and Post_Total). The subscores include the pre- and post-divergent creative thinking processes (Pre_Div and Post_Div) the pre- and post-convergent creative thinking processes (Pre_Conv and Post_Conv) the pre- and post-subscores of tasks with abstract stimuli (Pre_Abst and Post_Abst) and the pre- and post-subscores of tasks with concrete stimuli, (Pre_Conc and Post_Conc), called abstract and concrete creative thinking in the rest of the paper. Total EPoC scores represent the sum of divergent and convergent thinking subscores. The abstract and the concrete creative thinking processes scores were calculated summing the scores on abstract and concrete stimulation tasks.

**Table 2:** Descriptives of pre- and post-test EPoC subscores by group.

				95% CI	
	Subscores	*M*	*S.D.*	L.B.	U.B.
DA group	Pre_Total	10.51	0.46	9.60	11.42
	Post_Total	14.19	2.55	13.09	15.29
	Pre_Div	4.24	1.74	3.66	4.82
	Post_Div	5.76	2.42	4.95	6.56
	Pre_Conv	6.43	2.36	5.76	7.73
	Post_Conv	8.41	2.25	7.72	9.1
	Pre_Abst	5.41	0.26	4.88	5.93
	Post_Abst	7.16	0.30	6.57	7.76
	Pre_Conc	5.30	1.51	4.81	5.78
	Post_Conc	7.05	2.21	6.42	7.69
Control group	Pre_Total	10.23	0.38	9.47	10.99
	Post_Total	10.45	0.46	9.53	11.37
	Pre_Div	4.10	1.3	3.77	4.45
	Post_Div	3.70	2.0	3.16	4.24
	Pre_Conv	6.13	1.83	5.57	6.70
	Post_Conv	6.75	1.94	6.20	7.32
	Pre_Abst	4.74	0.22	4.30	5.17
	Post_Abst	5.64	0.25	5.14	6.14
	Pre_Conc	5.49	1.46	5.09	5.90
	Post_Conc	4.83	1.71	4.30	5.36

The correlations among all scores in the pre- and post-test of the DA group are presented in Table 3.

**Table 3:** Correlations in pre- and post-test EPoC scores by group.

		1	2	3	4	5	6	7	8	9	10
DA Group	1. Pre_Total	1									
	2. Post_Total	0.63**	1								
	3. Pre_Div	0.53**	0.07	1							
	4. Post_Div	0.49**	0.83**	0.07	1						
	5. Pre_Conv	0.71**	0.68**	-0.13	0.47**	1					
	6. Post_Conv	0.53**	0.81**	0.02	0.34*	0.66**	1				
	7. Pre_Abst	0.82**	0.47**	0.41*	0.29	0.69**	0.47**	1			
	8. Post_Abst	0.59**	0.91**	0.08	0.73**	0.59**	0.76**	0.41*	1		
	9. Pre_Conc	0.69**	0.59**	0.40*	0.44**	0.63**	0.54**	0.40*	50^∗∗^	1	
	10. Post_Conc	0.49**	0.90**	0.00	0.76**	0.66**	0.72**	0.41*	0.65**	0.60**	1
Control Group	1. Pre_Total	1									
	2. Post_Total	0.47**	1								
	3. Pre_Div	0.75**	0.38**	1							
	4. Post_Div	0.31*	0.78**	45^∗∗^	1						
	5. Pre_Conv	0.88**	0.32**	0.35*	0.12	1					
	6. Post_Conv	0.41**	0.77**	0.14	0.20	0.49**	1				
	7. Pre_Abst	0.87**	0.36**	0.67**	0.30*	0.75**	0.25	1			
	8. Post_Abst	0.40**	0.87**	0.33*	0.63**	0.34*	0.71**	0.34*	1		
	9. Pre_Conc	0.85**	0.45**	0.61**	0.23	0.77**	0.46**	0.47**	0.34*	1	
	10. Post_Conc	0.40**	0.87**	0.39*	0.73**	0.33*	0.62**	0.26	0.51**	0.43**	1

*Correlation significant for ^∗^*p* < 0.05 and ^∗∗^*p* < 0.01.*

It is worth noticing that the correlations between the pre- and post-test in most subscores are moderate.

### Effect of Mediation

In order to investigate the effect of mediation for the total score and the subscores of EPoC a series of analyses of variance (ANOVA) were carried out. First, the interactional effect of time × groups was investigated by a two-way mixed-design ANOVA having time (pre- and post-testing) as a within-subjects factor and the two groups of participants (DA group and control group) as a between-subjects factor. A series of Levene’s tests showed that the variances of the two groups were equal for both the pre-test [*F* (1,68) = 1.48, *p* > 0.05] and the post-test [*F* (1,68) = 2.82, *p* > 0.05] of total EPoC score, the pre-test [*F* (1,88) = 3.16, *p* > 0.05] and the post-test [*F* (1,88) = 1.91, *p* > 0.05] of divergent thinking, the pre-test [*F* (1.88) = 0.08, *p* > 0.05] and the post-test [*F* (1, 88) = 0.01, *p* > 0.05] of abstract thinking and the pre-test [*F* (1, 88) = 0.01, *p* > 0.05] and the post-test [*F* (1, 88) = 3.97, *p* = 0.05] of concrete thinking, while they were unequal for the pre-test [*F* (1, 88) = 4.76, *p* < 0.05] of convergent thinking but equal for the post-test [*F* (1,88) = 1.93, *p* > 0.05] of convergent thinking.

As seen in Table 4 the interactional effect of time by group was found to be statistically significant for the total score and for all EPoC subscores implying that that the mediation implemented on the DA group had a significant effect.

**Table 4:** Interaction effect of time × group in post-test.

Variable	*F*	*(df1,df2)*	*ω^2^*
Total	29.35***	(1, 88)	0.24
Divergent	15.01***	(1, 88)	0.13
Convergent	10.88**	(1, 88)	0.09
Abstract	4.27*	(1, 88)	0.04
Concrete	42.00***	(1, 88)	0.31

*Significant for ^∗^*p* < 0.05, ^∗∗^*p* < 0.01, and ^∗∗∗^*p* < 0.001.*

In order to explore the source of this interaction first we investigated the main effect of time on group separately, by the means of repeated measures analysis of variance (RM ANOVA) having pre- and post-scores as a within-subjects factor.

As seen in Table 5 for the DA group, the within subjects effect of time was significant for all measured variables. From the above analysis it appears that in total EPoC score, as well in the subscores of convergent, divergent, abstract and concrete thinking had a large effect. For the control group, the analysis showed that the subscores of post-test abstract and convergent thinking had a significant improvement over time, yet, with a small effect size, while a non-significant improvement over time was observed in total EPoC and divergent thinking. The subscores of divergent and concrete thinking had a drop in post-test compared with the pre-test (see Table 2). In sum, the results of this analysis demonstrated that mediation had a large significant effect on all subscores.

**Table 5:** Main within subject effect of time by group.

	Control group			DA group		
Variable	*F*	*(df1,df2)*	*ω^2^*	*F*	*(df1,df2)*	*ω^2^*
Total	0.32	(1,52)	0.01	53.54^∗∗∗^	(1, 36)	0.58
Divergent	2.57	(1,52)	0.02	10.27^∗∗^	(1, 36)	0.20
Convergen*t*	5.6*	(1,52)	0.07	39.59^∗∗∗^	(1, 36)	0.50
Abstract	12.25**	(1,52)	0.17	29,19^∗∗∗^	(1, 36)	0.43
Concrete	7.91**	(1,52)	0.11	0.35,81^∗∗∗^	(1, 36)	0.48

*Significant for ^∗^*p* < 0.05, ^∗∗^*p* < 0.01, and ^∗∗∗^*p* < 0.001.*

Finally, in order to investigate the main effect between the two groups univariate ANOVAs were conducted with pre-test scores as the dependent variables and group as the independent. Also, univariate ANCOVAs were carried out having the post-test score as the dependent variables, the two groups of participants as a fixed factor and pre-test as a covariant.

As demonstrated in Table 6 a non-significant between subjects effect between the two groups was observed in the pre-test scores of all measured variables, whereas a significant effect between the two groups was observed in the post-test of all variables. Specifically, it appears that in total score and concrete thinking there was a large effect size while a moderate effect size was found in divergent, convergent and abstract thinking. In conclusion, the two groups’ static scores as appear in the pre-test did not differ significantly while DA’s group post-test scores were significantly improved demonstrating the difference DA can make.

**Table 6:** Main between subjects effect of group by time.

	Pre-test			Post-test		
Variable	*F*	*(df1,df2)*	*ω^2^*	*F*	*(df1,df2)*	*ω^2^*
Total	0.23	(1, 88)	0.01	33.95^∗∗∗^	(1, 87)	0.27
Divergent	0.22	(1, 88)	0.00	19.75^∗∗∗^	(1, 87)	0.18
Convergent	0.46	(1, 88)	0.06	16.20^∗∗∗^	(1, 87)	0.15
Abstract	3.82	(1, 88)	0.03	10.98^∗∗^	(1, 88)	0.13
Concrete	0.37	(1, 88)	0.00	28.79^∗∗∗^	(1, 88)	0.32

*Significant for ^∗∗^*p* < 0.01, and ^∗∗∗^*p* < 0.001.*

## Discussion

In general, according to the results, DA appeared to be a better indicator of students CP which can assist subsequent decisions in instruction. In particular, the correlations among subscores reflected the construct validity of EPoC and the correlations between test and retest supported the reliability of EPoC (Barbot et al., 2015). Nevertheless, the finding that the pre- and post-test correlations of the DA group were not very strong and the variance explained was from 4.9 to 43.5% indicated that mediation effect varied among students in other words not all students gained the same benefit from DA.

Overall, the ANOVAs demonstrated the effectiveness of mediation. The non-significant differences between the groups in pre-testing showed that the two groups were of equal CP at the starting point of the study. The significant but small effect size of time on the control group’s post-test total score as well as the disparity in the effect of time on the individual subscores implied that the pre-testing experience may have had a limited impact in the control group’s post-test scores. However, the most important finding of the study was the significant effect of mediation on all variables examined. This is in line with past research regarding inductive (analogical) reasoning and problem solving (e.g., Wang, 2010; Resing et al., 2017a,b). The present study provided evidence that personalized instruction and interaction between the assessor and the assesse may enhance not only the development of children’s inductive reasoning but also their creative thinking skills.

The reason for the large effect sizes of improvement needs further consideration. Previous studies in other cognitive domains have demonstrated effect sizes (eta squared) that varied between 0.10 to 0.40 (e.g., Vogelaar and Resing, 2016; Resing et al., 2017b). Given the limited time length of the mediation, such effect sizes are hardly likely to demonstrate any major developments in creative thinking abilities. It is more likely that the effect sizes reflect improvement in children’s understanding regarding the task demands along with the development of strategies for expressing the required cognitive abilities, as well as an enhancement of motivation related constructs such as creative self-efficacy. The magnitude of the effect size may also be indicative of Greek students’ lack of experience in creative activities and testing. As mentioned in the sample description, the present sample consisted of primary school children from a socially deprived area of Athens inner city. It is possible that these young students probably had never been asked to employ divergent thinking or to produce novel ideas, or to have received any feedback on such efforts. In this regard it is likely that they had not fully understood the demands of the tasks and the required cognitive strategies nor they had been motivated to engage regardless the accuracy of the test instructions. It seems therefore that DA of CP could be helpful for revealing the CP of disadvantaged young students, confirming in the area of creativity the finding by numerous previous studies that demonstrated the benefits for disadvantaged students from the application of DA in various cognitive domains (e.g., Resing et al., 2009, 2017b; Navarro and Lara, 2017).

### Implications for Practice

Despite its importance, creativity development in schools still remains an unachieved educational goal (e.g., Beghetto and Kaufman, 2014) partially due to the lack of an assessment method that identifies students’ CP and facilitates in setting the aims and the content of specific educational interventions and teaching decision making towards creativity development (Kaufman et al., 2009; Barbot et al., 2015). For this purpose, the static use of EPoC allows the creation of students’ CP personal profiles which can be of great assistance for assessing and nurturing creativity. In the light of the findings of the present study, we argue that students’ personal profiles based on the results obtained from DA of CP would be more informative for designing educational programs aiming at creativity nurturing, especially for children from disadvantaged backgrounds. That is so, because conclusions based on the results of static measurement may be misleading for educators, as they may interpret low student scores in creativity as lack of or low CP and consequently trigger the vicious circle of low expectations which may lead to low achievements and so on.

Furthermore, students may interpret low scores as lack of creative ability which could in turn result into low creative self-efficacy, low goals setting and into maladaptive motivation in general. A profile of CP created with DA of CP using EPoC would offer much more informed and beneficial facts for use by teachers for the nurturing of creativity. A profile of CP which includes the development in creativity dimensions together with the experience of the interaction between the assessor and the assessee during the process of mediation, would provide qualitative information about a series of factors that may had hindered the expression of CP such as misunderstandings, motivation, etc.

### Limitations and Directions for Future Research

The present study can only be characterized as preliminary in the study of DA for the assessment of CP. Due to the limited size of the sample the results cannot be generalized for the student population and further research is needed with more students from different cultural and educational backgrounds as well as students of different age or cognitive ability. The data collected did not allow conclusions about the characteristics of the students that benefitted more from mediation, which needs further research. Certainly further research should be conducted in order to investigate the effect of DA in other domains such as linguistic or mathematical creativity. Moreover, the transfer of improvement on creative thinking in one domain of creativity into different domains needs to be studied. In addition, it would be useful to investigate DA using other CP tests to examine the effect of mediation on them. Finally, an “active control group” besides control group can be used in the semi-experimental design in the future. The “active control group” could receive a mediation aiming at developing a different, unrelated to creativity cognitive ability such as memory. Comparisons among post-scores of all groups would provide more valid information for the effectiveness of mediation in enhancing creativity.

Besides its limitations, we believe that the importance of our study lies in its implications for creativity assessment and real classroom practice. As this study demonstrated, static assessment of CP cannot fully depict children’s CP while DA provides a clearer portrait which can be utilized especially in education.

## Author Contributions

Both authors contributed substantially to the conception or design of the work, the acquisition, analysis or interpretation of data for the work as well as to draft the work or revise it critically for important intellectual content. In addition they both provided approval for publication of the content and agreed to be accountable for all aspects of the work in ensuring that questions related to the accuracy or integrity of any part of the work are appropriately investigated and resolved.

## Conflict of Interest Statement

The authors declare that the research was conducted in the absence of any commercial or financial relationships that could be construed as a potential conflict of interest.
